# Unexpected role of microglia and P2Y_12_ in the induction of and emergence from anesthesia

**DOI:** 10.1007/s11302-024-10014-1

**Published:** 2024-05-10

**Authors:** Bijay Parajuli, Schuichi Koizumi

**Affiliations:** 1 Department of Neuropharmacology, Interdisciplinary Graduate School of Medicine, University of Yamanashi, 1110 Shimokato, Chuo, Yamanashi, 409-3898 Japan; 2 GLIA Center, University of Yamanashi, Yamanashi, 409-3898 Japan

## Article summary 

General anesthetics act on neurons to cause overall suppression of neuronal activity in the brain leading to loss of consciousness that renders a patient unarousable to painful stimuli. General anesthesia is reported to act on various ion channels, such as GABA_A_, glycine, nicotinic acetylcholine, and NMDA receptors expressed on neurons [1]. Typically, general anesthetics potentiate the activation of inhibitory postsynaptic channels or inhibit the activation of excitatory synaptic channels. In a recent publication in eLife, He et al., have shown that microglia, immune cell in the central nervous system (CNS), play an essential role in the sensitivity of mice to anesthetics [2]. They found that microglia-depleted mice required a longer time for loss of righting reflex (LORR) and a shorter time for recovery of righting reflex (RORR) induced by multiple anesthetics indicating that microglia are required for the induction and maintenance of anesthesia. Microglial repopulation reversed these effects. They further showed that genetic knockout or pharmacological inhibition of microglial P2Y_12_ or receptors or the downstream rise in cytoplasmic Ca^2+^ level, rendered mice more resistant to general anesthesia. The study highlights the essential role of microglia in the induction and emergence of anesthetics and expands the non-immune function of microglia in brain.

## Commentary

Communication between neurons occurs at tiny gaps called synapses, a specialized area where the presynaptic and postsynaptic neurons come within nanometers of one another to allow for signal transmission. The presynaptic neuron releases a neurotransmitter that is received by the postsynaptic neuron’s neurotransmitter receptors. Neurotransmitters act on two types of neurotransmitter receptors, ligand-gated ion channels (LGIC) and G-protein–coupled receptors (GPCR). Depending on their functions, neurotransmitters are classified as excitatory neurotransmitters or inhibitory neurotransmitters. Excitatory neurotransmitters, for example glutamate and acetylcholine, act on LGIC receptors to induce the flow of cations (mainly Na^+^) across the plasma membrane, causing the membrane potential to depolarize, bringing it nearer to the action potential threshold. Conversely, inhibitory neurotransmitters, such as GABA, act on LGIC receptors to induce the flow of anions (mainly Cl^−^) to hyperpolarize the membrane potential. It is thought that the action of anesthetics and the various behavioral response patterns that they induce are due to the modulation of LGIC in neuron [1, 3, 4]. Typically, anesthetics potentiate the activation of inhibitory LGIC or inhibit the activation of excitatory LGIC to induce unconsciousness.

Microglia are the innate immune cells of CNS and constitute about 10–15% of all glial cells [5]. They derive from the primitive yolk sac, populate the brain rudiment of the developing embryo by embryonic day 8 (E8) and are embryologically distinct from the monocyte/macrophage system [6]. These cells participate in CNS development, homeostasis, and serve as an important connection between neurological and immunological activity in the CNS. Microglia constantly survey their surrounding microenvironment through the extension and retraction of their highly motile processes, interact with neurons and are involved in control of neuronal activity [7, 8].

Previous studies have shown that microglial surveillance is increased in anesthetized mice [9, 10]. In this report He et al., found that that microglia play essential role in the sensitivity of mice to anesthetics. Using behavioral (LORR and RORR) and electrophysiological (EEG and EMG) approaches, they showed that when microglia are depleted using CSF1R antagonist, PLX5622, mice exhibited delayed anesthesia induction and early emergence from anesthesia induced by a GABA_A_ receptor agonist or a NMDA receptor antagonist [2]. They also showed that microglial repopulation restored the times taken for LORR and RORR induced by anesthetics. PLX5622 can ablate both brain microglia and peripheral macrophages [11]. To determine which of these cells is responsible for the observed effects, they used the blood–brain barrier-impermeable CSF1R inhibitor, PLX73086, to ablate peripheral macrophages without affecting brain microglia and found that it had no effect on the sensitivity to anesthetics indicating that the depletion of microglia is essential for the observed effect. In general, using multiple experimental approaches, they uncovered the essential role of microglia in facilitating and stabilizing the general anesthesia response.

Different brain regions regulate anesthesia induction and emergence from anesthesia [3, 4]. Based on neuronal c-Fos upregulation using immunohistochemistry and RNA in-situ hybridization, they showed that microglial depletion decreased neuronal activity in two regions associated with induction of general anesthesia, lateral habenula and supraoptic nucleus (SON) and increased neuronal activity in two regions associated with emergence of paraventricular thalamus, locus coeruleus (LC). They substantiated their immunohistochemistry findings with electrophysiological experiments and found that the excitatory/inhibitory (E/I) ratio was also significantly decreased in the SON of microglia-depleted mice, indicating decreased neuronal excitability. Furthermore, they also found that the E/I ratio in the LC was significantly enhanced, indicating increased neuronal excitability. Their results indicate that microglia both negatively and positively regulate neuronal activity, depending on the brain regions studied.

The metabotropic P2Y_12_ receptor is expressed in microglia, but not macrophages and purinergic signaling is a key mechanism that regulates dynamic interactions between neurons and microglia [12, 13]. In the CNS, ATP released from neurons is enzymatically degraded to ADP and activates microglial P2Y_12_ receptors [14]. To investigate the underlying mechanism, He et al., used pharmacological inhibition and genetic deletion of microglial P2Y_12_ receptors. They found that both methods delayed LORR and accelerated RORR. To further validate this finding, they replaced microglia with bone marrow-derived cells that mature into P2Y_12_ receptor-negative microglia-like cells. These microglia replaced mice showed similar outcomes to those observed after P2Y_12_ receptor-knockout. The P2Y_12_ receptor is a GPCR and its activation leads to a rise in the concentration of Ca^2+^ inside microglia [15, 16]. To further determine whether microglia-mediated modulation of the anesthesia response depends on downstream Ca^2+^ signaling, they utilized designer receptors exclusively activated by designer drugs (DREAADs) to selectively elevate the intracellular Ca^2+^ level in microglia. Under these conditions, they found that LORR was accelerated and RORR was delayed. Further, they genetically deleted STIM1, an endoplasmic reticulum Ca^2+^ sensor, in microglia to disrupt intracellular Ca^2+^ signaling and found that this was sufficient to delay LORR and accelerate RORR. These data convincingly show that microglial purinergic receptor and intracellular Ca^2+^ signaling, are essential for regulating anesthesia in mice. However, the molecular mechanism through which microglial Ca^2+^ affects neuronal activity remains unknown. Alongside another recent publication which also revealed that microglia modulate response to general anesthesia through P2Y_12_ receptors [17], the findings of He et al., provide a new perspective of how microglia regulate anesthesia.

Although the authors have not provided evidence for how microglia control neuronal activity, it has also recently been shown that microglial depletion enhances neuronal activity in the striatum. ATP released by neuron triggers microglial adenosine production, which regulates neuronal responses via neuronal adenosine A_1_ receptor [18]. A similar mechanism may be responsible for the observed increased neuronal activity in the LC of microglia-depleted mice. Additionally, in a recent publication, Haruwaka et al., reported that microglia transiently increase neuronal activity, after cessation of isoflurane anesthesia by shielding axosomatic GABAergic synapses onto excitatory neurons during the anesthesia phase thereby decreasing inhibitory inputs to excitatory neurons in the somatosensory cortex [19]. A similar mechanism maybe responsible for decreased neuronal activity in the SON of microglia depleted mice.

To conclude He et al., showed that microglia play an essential role in the sensitivity of mice to anesthetics. Microglia are activated in various neurodegenerative disorders and in aged mice. Further investigation studying the effect of anesthesia induction and post anesthesia recovery in mice with activated microglia can lead to a more comprehensive understanding of the how communication between neurons and microglia within the brain occurs in physiological and pathophysiological conditions and will have important clinical implications.

## Data Availability

Not applicable.

## References

[1] Franks NP (2008) General anaesthesia: from molecular targets to neuronal pathways of sleep and arousal. Nat Rev Neurosci 9:370–386. 10.1038/nrn2372 18425091 10.1038/nrn2372

[2] He Y et al (2023) Microglia facilitate and stabilize the response to general anesthesia via modulating the neuronal network in a brain region-specific manner. Elife 12. 10.7554/eLife.92252 10.7554/eLife.92252 PMC10746144 38131301

[3] Lyu J, Cai H, Chen Y, Chen G (2022) Brain areas modulation in consciousness during sevoflurane anesthesia. Front Integr Neurosci 16:1031613. 10.3389/fnint.2022.1031613 36619239 10.3389/fnint.2022.1031613 PMC9811387

[4] Kelz MB, Garcia PS, Mashour GA, Solt K (2019) Escape from oblivion: neural mechanisms of emergence from general anesthesia. Anesth Analg 128:726–736. 10.1213/ANE.0000000000004006 30883418 10.1213/ANE.0000000000004006 PMC6885009

[5] Hanisch UK, Kettenmann H (2007) Microglia: active sensor and versatile effector cells in the normal and pathologic brain. Nat Neurosci 10:1387–1394. 10.1038/nn1997 17965659 10.1038/nn1997

[6] Ginhoux F et al (2010) Fate mapping analysis reveals that adult microglia derive from primitive macrophages. Science 330:841–845. 10.1126/science.1194637 20966214 10.1126/science.1194637 PMC3719181

[7] Nimmerjahn A, Kirchhoff F, Helmchen F (2005) Resting microglial cells are highly dynamic surveillants of brain parenchyma in vivo. Science 308:1314–1318. 10.1126/science.1110647 15831717 10.1126/science.1110647

[8] Wake H, Moorhouse AJ, Jinno S, Kohsaka S, Nabekura J (2009) Resting microglia directly monitor the functional state of synapses in vivo and determine the fate of ischemic terminals. J Neurosci 29:3974–3980. 10.1523/JNEUROSCI.4363-08.2009 19339593 10.1523/JNEUROSCI.4363-08.2009 PMC6665392

[9] Stowell RD et al (2019) Noradrenergic signaling in the wakeful state inhibits microglial surveillance and synaptic plasticity in the mouse visual cortex. Nat Neurosci 22:1782–1792. 10.1038/s41593-019-0514-0 31636451 10.1038/s41593-019-0514-0 PMC6875777

[10] Liu YU et al (2019) Neuronal network activity controls microglial process surveillance in awake mice via norepinephrine signaling. Nat Neurosci 22:1771–1781. 10.1038/s41593-019-0511-3 31636449 10.1038/s41593-019-0511-3 PMC6858573

[11] Yang X et al (2020) CSF1R blockade induces macrophage ablation and results in mouse choroidal vascular atrophy and RPE disorganization. Elife 9. 10.7554/eLife.55564 10.7554/eLife.55564 PMC7156269 32234210

[12] Sasaki Y et al (2003) Selective expression of Gi/o-coupled ATP receptor P2Y12 in microglia in rat brain. Glia 44:242–250. 10.1002/glia.10293 14603465 10.1002/glia.10293

[13] Lin SS, Tang Y, Illes P, Verkhratsky A (2020) The safeguarding microglia: central role for P2Y(12) receptors. Front Pharmacol 11:627760. 10.3389/fphar.2020.627760 33519493 10.3389/fphar.2020.627760 PMC7840491

[14] Zimmermann H, Zebisch M, Strater N (2012) Cellular function and molecular structure of ecto-nucleotidases. Purinergic Signal 8:437–502. 10.1007/s11302-012-9309-4 22555564 10.1007/s11302-012-9309-4 PMC3360096

[15] Saito K et al (2024) Microglia sense astrocyte dysfunction and prevent disease progression in an Alexander disease model. Brain 147:698–716. 10.1093/brain/awad358 37955589 10.1093/brain/awad358 PMC10834242

[16] Jiang P et al (2017) Nucleotide transmitters ATP and ADP mediate intercellular calcium wave communication via P2Y12/13 receptors among BV-2 microglia. PLoS ONE 12:e0183114. 10.1371/journal.pone.0183114 28800362 10.1371/journal.pone.0183114 PMC5553643

[17] Cao K et al (2023) Microglia modulate general anesthesia through P2Y(12) receptor. Curr Biol 33:2187-2200 e2186. 10.1016/j.cub.2023.04.047 37167975 10.1016/j.cub.2023.04.047

[18] Badimon A et al (2020) Negative feedback control of neuronal activity by microglia. Nature 586:417–423. 10.1038/s41586-020-2777-8 32999463 10.1038/s41586-020-2777-8 PMC7577179

[19] Haruwaka K et al (2024) Microglia enhance post-anesthesia neuronal activity by shielding inhibitory synapses. Nat Neurosci. 10.1038/s41593-023-01537-8 38177340 10.1038/s41593-023-01537-8 PMC10960525

